# Chinese Herbal Mixture, Tien-Hsien Liquid, Induces G2/M Cycle Arrest and Radiosensitivity in MCF-7 Human Breast Cancer Cells through Mechanisms Involving DNMT1 and Rad51 Downregulation

**DOI:** 10.1155/2016/3251046

**Published:** 2016-07-20

**Authors:** Chih-Jung Yao, Jyh-Ming Chow, Chia-Ming Yang, Hui-Ching Kuo, Chia-Lun Chang, Hsin-Lun Lee, I-Chun Lai, Shuang-En Chuang, Gi-Ming Lai

**Affiliations:** ^1^Comprehensive Cancer Center, Taipei Medical University, Taipei 11031, Taiwan; ^2^Cancer Center, Wan Fang Hospital, Taipei Medical University, Taipei 11696, Taiwan; ^3^Department of Internal Medicine, School of Medicine, College of Medicine, Taipei Medical University, Taipei 11031, Taiwan; ^4^Division of Hematology and Medical Oncology, Department of Internal Medicine, Wan Fang Hospital, Taipei Medical University, Taipei 11696, Taiwan; ^5^Formosa Cancer Foundation, Taipei 10597, Taiwan; ^6^The Ph.D. Program for Translational Medicine, Taipei Medical University-Academia Sinica, Taipei 11031, Taiwan; ^7^Division of Radiation Oncology, Department of Oncology, Taipei Veterans General Hospital, Taipei 11217, Taiwan; ^8^National Institute of Cancer Research, National Health Research Institutes, Miaoli County 35053, Taiwan

## Abstract

The Chinese herbal mixture, Tien-Hsien Liquid (THL), has been proven to suppress the growth and invasiveness of cancer cells and is currently regarded as a complementary medicine for the treatment of cancer. Our previous study using acute promyelocytic leukemia cells uncovered its effect on the downregulation of DNA methyltransferase 1 (DNMT1) which is often overexpressed in cancer cells resulting in the repression of tumor suppressors via hypermethylation. Herein, we explored the effects of THL in MCF-7 breast cancer cells that also demonstrate elevated DNMT1. The results show that THL dose-dependently downregulated DNMT1 accompanied by the induction of tumor suppressors such as p21 and p15. THL arrested cell cycle in G2/M phase and decreased the protein levels of cyclin A, cyclin B1, phospho-pRb, and AKT. DNMT1 inhibition was previously reported to exert a radiosensitizing effect in cancer cells through the repression of DNA repair. We found that THL enhanced radiation-induced clonogenic cell death in MCF-7 cells and decreased the level of DNA double-strand break repair protein, Rad51. Our observations may be the result of DNMT1 downregulation. Due to the fact that DNMT1 inhibition is now a mainstream strategy for anticancer therapy, further clinical trials of THL to confirm its clinical efficacy are warranted.

## 1. Introduction

The current mainstream modalities for cancer therapy, such as surgery, chemotherapy, and radiotherapy, often result in unsatisfactory efficacy and adverse side effects. Patients resort to complementary and alternative medicines for improving clinical outcomes. In this regard, Chinese herbal medicine represents a viable resource. One potential candidate is the Chinese herbal mixture, Tien-Hsien Liquid (THL), which has been used as a complementary and alternative herbal medicine for cancer for over twenty years [1]. The herbal mixture consists of extracts from 14 Chinese medicinal herbs:* Cordyceps sinensis* (CS),* Oldenlandia diffusa* (OD),* Indigo Pulverata Levis* (IPL, also known as indigo naturalis),* Polyporus umbellatus* (PU),* Radix Astragali* (RA),* Panax ginseng* (PG),* Solanum nigrum* L. (SNL),* Pogostemon cablin* (PC),* Atractylodis Macrocephalae Rhizoma* (AMR),* Trichosanthes Radix* (TR),* Clematis Radix* (CR),* Margarite *(M),* Ligustrum lucidum* Ait. (LLA), and* Glycyrrhizae Radix* (GR) [2]. In 2012, the safety and efficacy of THL in immune system augmentation were demonstrated in a randomized, double-blind, placebo-controlled, parallel-group, phase IIa clinical trial for patients with refractory metastatic breast cancer [1].

The anticancer activities of THL include induction of apoptosis in cancer cells [3], modulation of immune cells [2], inhibition of angiogenesis and metastasis, and suppression of tumor growth in animal model [4]. In addition to the inhibition of PML-RAR*α* oncogenic fusion protein, our previous study in NB4 human acute promyelocytic leukemia (APL) cells also uncovered the effect of THL on repressing DNMT1 (DNA methyltransferase 1) protein [5], which is often abnormally upregulated in cancer cells resulting in the suppression of tumor suppressor genes by hypermethylation [6].

Methylation of CpG-rich promoter regions at the 5-position of cytosine by DNMTs can epigenetically repress the expression of genes. Among the three known DNMTs (DNMT1, DNMT3A, and DNMT3B), DNMT1 is the most abundant and well-studied. DNMT1 is responsible for the maintenance of methylation across successive cell generations [6, 7]. It methylates newly biosynthesized DNA and is associated with the replication machinery, while DNMT3A and DNMT3B function as de novo methyltransferases that add methyl groups to bare DNA [6, 8]. As DNMT1 is the most abundant methyltransferase in dividing cells and occurs at lower levels in nondividing cells, it has become the major target for methylation inhibition in rapidly dividing cancer cells [9]. Therefore, DNMT1 inhibition is an important potential approach for cancer treatment [6, 10].

A clinicopathologic study showed that the level of DNMT1 is significantly higher in sporadic breast cancer tissue than in breast fibroadenoma [11]. This important observation provides validity for a novel concept and strategy in the treatment of breast cancer via the targeting of DNMT1. Vijayaraghavalu et al. demonstrated that inhibiting DNMT1 in MCF-7 breast cancer cells by 5-aza-2′ deoxycytidine (decitabine), a DNMT inhibitor, results in the induction of tumor suppressor p21 and G2/M cycle arrest [12]. As an extension of the observation that THL profoundly represses DNMT1 protein level in APL cells [5], we explored the effects of THL on MCF-7 human breast cancer cells possessing aberrantly elevated DNMT1 protein [7] in an attempt to elucidate its anticancer activities in association with DNMT1 downregulation. Our results showed that THL also diminished the expression of DNMT1 in MCF-7 cells, and this was accompanied by the induction of p21 and cell cycle arrest in the G2/M phase.

DNMT1 inhibition has also been reported to sensitize various types of cancer cells to radiation [8, 13, 14] and the mechanism was proposed to correlate with the inhibition of DNA double-strand break (DSB) repair [13]. When the radiosensitivity of THL-treated MCF-7 cells was investigated, we observed that THL augmented the radiation-mediated suppression of clonogenicity and this was associated with a reduction in the level of DNA DSB repair protein, Rad51. In summary, our results demonstrate that THL causes G2/M cycle arrest and promote radiosensitivity in human breast cancer cells. The possible mechanisms corresponding to these effects are proposed to involve tumor suppressor induction and DNA DSB repair repression orchestrated by DNMT1 inhibition, respectively.

## 2. Materials and Methods

### 2.1. Culture of Cells

MCF-7 (luminal A), MDA-MB-231 (triple-negative), and BT-474 (luminal B) human breast carcinoma cells and HS68 human foreskin fibroblast cells were obtained from the Bioresource Collection and Research Center (BCRC, Food Industry Research and Development Institute, Hsinchu, Taiwan). MCF-7, MDA-MB-231, and HS68 cells were cultured in Dulbecco's Modified Eagle's Medium (DMEM) (Invitrogen Life Technologies, CA, USA) and BT-474 cells were cultured in RPMI (Invitrogen). All media used contained 10% fetal bovine serum (FBS) and 1% penicillin/streptomycin (Invitrogen). The cells were incubated at 37°C in an atmosphere containing 5% CO_2_.

### 2.2. Reagents

THL was provided by Feida Union Pharmaceutical Manufactory, El Monte, CA. It is an aqueous preparation of herbal mixture and consists mainly of extracts from 14 Chinese medicinal herbs as mentioned previously. The high-performance liquid chromatography (HPLC) fingerprint of THL and the consistency between different batches have been demonstrated in the recent study by Leigh et al. [15]. The aqueous THL preparation was lyophilized, weighed, and then stored at −20°C. The lyophilized powder was reconstituted with sterile distilled water and added to cells to achieve the final test concentrations. Sulforhodamine B (SRB) for cell viability assays and Amido black for cell colony staining were purchased from Sigma (St. Louis, MO, USA).

### 2.3. Cellular Viability

MCF-7, MDA-MB-231, and BT-474 cells were seeded into 96-well plates at a density of 2.5 × 10^3^ cells/well. On the day after seeding, cells were treated with PBS (control) or various concentrations (0.75, 1.5, 3, and 6 mg/mL) of THL for 3 days. Briefly, treated cells were fixed in 10% trichloroacetic acid and then stained with 0.4% SRB. After incubation and washing, bound SRB was solubilized in 100 *μ*L of 10 mM unbuffered Tris base and the optical density was measured at 562 nm using a microtiter plate reader (Molecular Devices, Sunnyvale, CA, USA) [16, 17].

### 2.4. Analysis of the Cell Cycle

Twenty-four hours after being seeded into the wells of a 6-well plate, MCF-7, MDA-MB-231, and BT-474 cells (2 × 10^5^ cells/well) were treated with THL (0.75, 1.5, 3, and 6 mg/mL) or PBS control for 72 h. On the day of harvest, cells were fixed in ice-cold 70% ethanol and stored at −20°C. Cells were then washed twice with ice-cold phosphate-buffered saline (PBS) and incubated with RNase and DNA intercalating dye propidium iodide (50 *μ*g/mL). The cell cycle distribution was then analyzed by flow cytometry (BD Biosciences, San Jose, CA, USA).

### 2.5. Western Blot Analysis and Antibodies

MCF-7, BT-474, and HS68 cells were seeded into 10 cm dishes at a density of 10^6^ cells/dish and incubated for 24 h before being treated with THL (0.375, 0.75, 1.5, 3, and 5 mg/mL) or PBS control for 3 days. On the day of harvest, whole-cell lysates were prepared with buffer containing 50 mM Tris-HCl (pH 7.4), 150 mM NaCl, 1% Nonidet P40, 0.25% sodium deoxycholate, 1 mM ethylenediaminetetraacetic acid (EDTA), 1 mM Na_3_VO_4_, 1 mM NaF, 1 mM phenylmethanesulfonyl fluoride, and 1 : 100 diluted protease inhibitors mixture (Sigma, St. Louis, MO, USA). The protein extracts were subjected to SDS polyacrylamide gel electrophoresis and transferred to polyvinylidene difluoride membrane (Bio-Rad, Richmond, CA, USA) by electroblotting. The membranes were blocked with 5% BSA in 1x TBST (25 mM Tris-HCl, 125 mM NaCl, and 0.1% Tween 20) for 1 hour at room temperature and probed with primary antibody overnight at 4°C followed by horseradish peroxidase-conjugated secondary antibody for 1 hour at room temperature. Bands were visualized using Luminol reagent (Santa Cruz Biotechnology, Santa Cruz, CA, USA) according to the manufacturer's instructions. Primary antibodies against cyclin A (sc-239), cyclin B1 (sc-245), Cdc2 (sc-54), DNMT1 (sc-20701), DMNT3A (sc-20703), ERK (sc-94), phospho-ERK (p-ERK, sc-7383), Ku70 (sc-17789), Ku86 (sc-9034), and Rad51 (sc-8349) were purchased from Santa Cruz Biotechnology (Santa Cruz, CA, USA). Primary antibodies against AKT (#9272), phospho-AKT (p-AKT, #9271), and phospho-Rb (#9308) were purchased from Cell Signaling Technology (Beverly, MA, USA). Primary antibodies against actin (A5441), tubulin (T5168), and GAPDH (G8795) were purchased from Sigma (St. Louis, MO, USA).

### 2.6. Irradiation

MCF-7 breast cancer cells were seeded onto 6-well plates at a density of 200 cells per well. The following day the cells were treated with THL (0.375 and 0.75 *µ*g/mL) for 24 h after which they were irradiated by a Cesium-137 source (CISBIO International, irradiator model IBL 637) at doses of 4, 6, 12, and 24 Gy. Colony formation of the irradiated cells was assayed after 8 days of incubation. The response of MCF-7 cells to ionizing radiation was assessed by the colony formation assay. The sensitizer enhancement ratio was calculated (at a survival fraction of 50%) as the radiation dose needed for radiation-alone treatments divided by the radiation dose needed for THL-plus-radiation treatments.

### 2.7. Colony Formation Assay

MCF-7 breast cancer cells were seeded onto 6-well plates at a density of 200 cells per well. Eight days after treatment, colonies were stained with Amido black and the number of colonies containing at least 50 cells was determined for calculating the surviving fraction.

### 2.8. Statistical Analysis

Cell viability and colony formation data (mean ± SE in triplicate) were expressed as a percentage of the PBS control. In Figure 6(a), differences between groups were evaluated by one-way ANOVA followed by Dunnett's *t*-test. A difference at *p* < 0.05 was considered statistically significant.

## 3. Results

### 3.1. The SRB Assay Was Performed to Measure the Viability of MCF-7, MDA-MB-231, and BT-474 Cells Exposed to THL for 72 h

**Figure 1:**
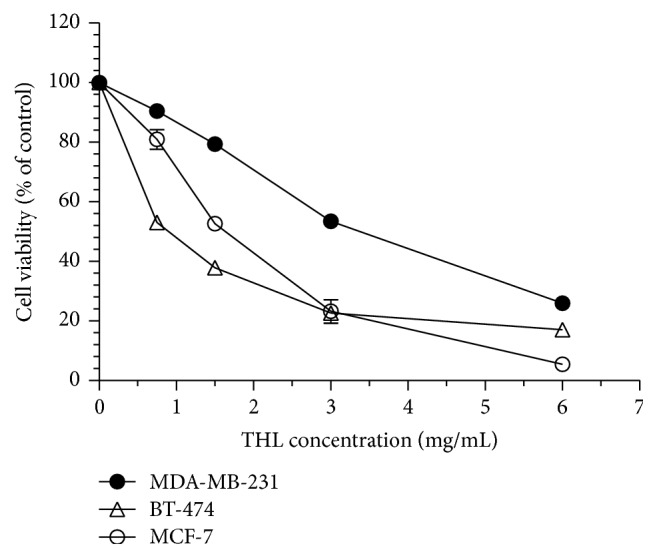
THL inhibited the growth of MCF-7, MDA-MB-231, and BT-474 breast cancer cells. Cells were treated with various concentrations of THL (0.75–6 mg/mL) or PBS only as a control for 72 h and cell viability was determined by the SRB assay. Data (mean ± SE in triplicate) are expressed as a percentage compared to the PBS-treated control.

**Figure 2:**
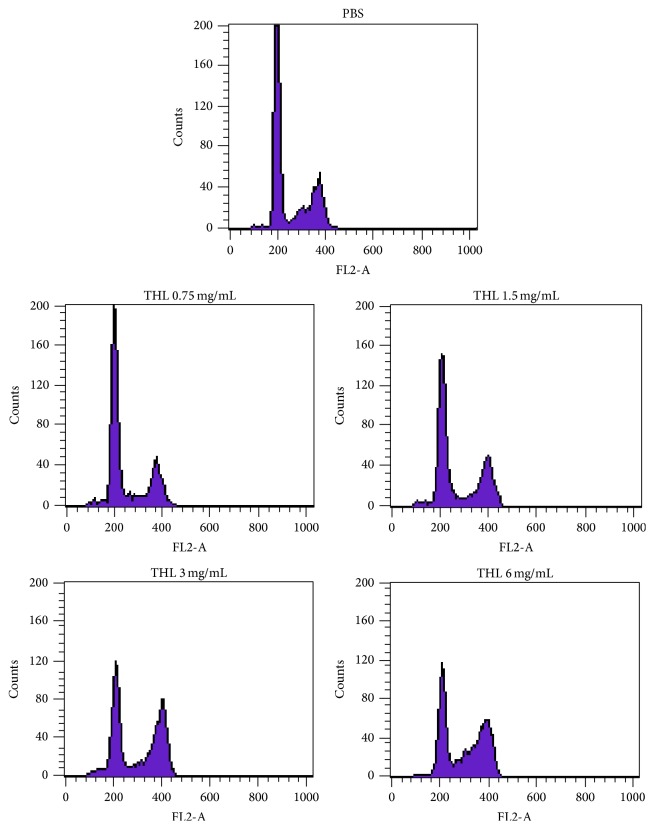
THL arrested the cell cycle of MCF-7 cells in G2/M phase. MCF-7 cells were treated with increasing concentrations of THL (0.75–6 mg/mL) or PBS only, for 72 h, and the cell cycle distribution was analyzed by flow cytometry. THL increased the percentage of G2/M phase in a dose-dependent manner. The percentage of cells in different phases of the cell cycle is shown in Table 1.

As shown in Figure 1, THL decreased the cellular viability of the three types of breast cancer cells in a dose-dependent manner. IC_50_ of THL in MCF-7, BT-474, and MDA-MB-231 cells were 1.6, 0.75, and 3 mg/mL, respectively. Flow cytometric analysis of the MCF-7 cell cycle revealed a dose-dependent increase in the G2/M fraction after treatment with THL compared to the PBS-treated control (Figure 2). The percentage of MCF-7 cells arrested at G2/M phase increased from 22.95% (PBS control) to 24.75%, 33.41%, 44.33%, and 45.84% at THL doses of 0.75, 1.5, 3.0, and 6.0 mg/mL, respectively (Table 1). Similar results were obtained for THL-treated MDA-MB-231 cells (Table 2). In contrast, THL induced a profound increase in the SubG1 fraction in BT-474 cells accompanied by S-phase arrest (Table 3).

### 3.2. THL Decreases the Level of Cyclin A and Cyclin B1 in MCF-7 Breast Cancer Cells

**Figure 3:**
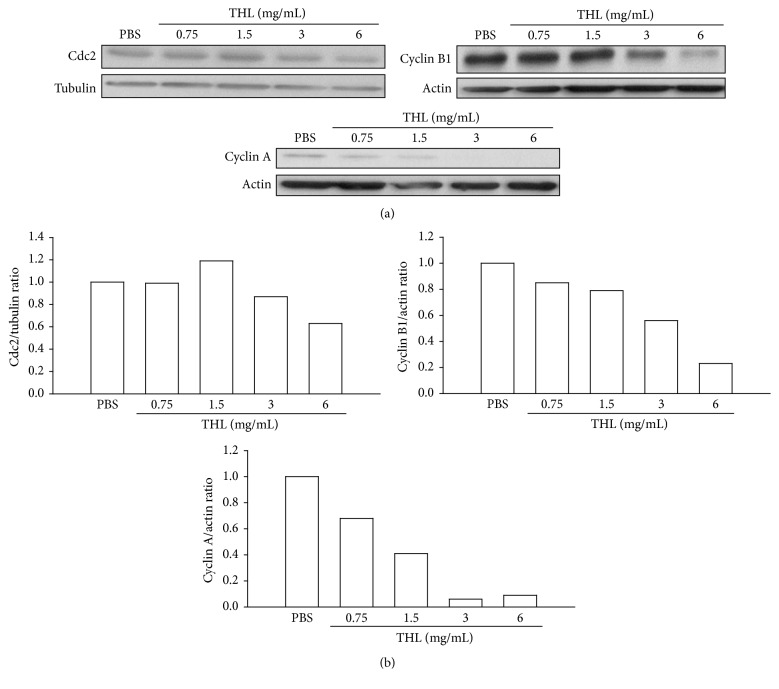
THL decreased G2/M-related cyclins A and B1 in MCF-7 cells. (a) THL decreased cyclin A and cyclin B1 protein levels in a dose-dependent manner but did not significantly change the Cdc2 (CDK1) protein level. Cells were treated with PBS or THL (0.75, 1.5, 3, and 6 mg/mL) for 72 h and then harvested. Cell lysates were then prepared for Western blot analysis. (b) The blots were normalized to actin or tubulin, and the fold change in protein expression levels in comparison to the PBS-treated control is depicted in this panel.

 In accordance with G2/M phase arrest, the associated cyclins required for passage through the G2/M phase such as cyclin A and cyclin B1 proteins [18, 19] were observed to decrease as the dose of THL increased (Figure 3). In contrast, the protein level of G2/M cyclin-dependent kinase (CDK) Cdc2 (cell division cycle protein 2, CDK1) was not significantly reduced even at the highest dose of THL tested (Figure 3). We then examined whether THL induced expression of CDK inhibitor such as p21, which suppresses Cdc2 activity [20].

### 3.3. THL Downregulates DNMT1 and Induces p21 and p15 in MCF-7 Breast Cancer Cells

**Figure 4:**
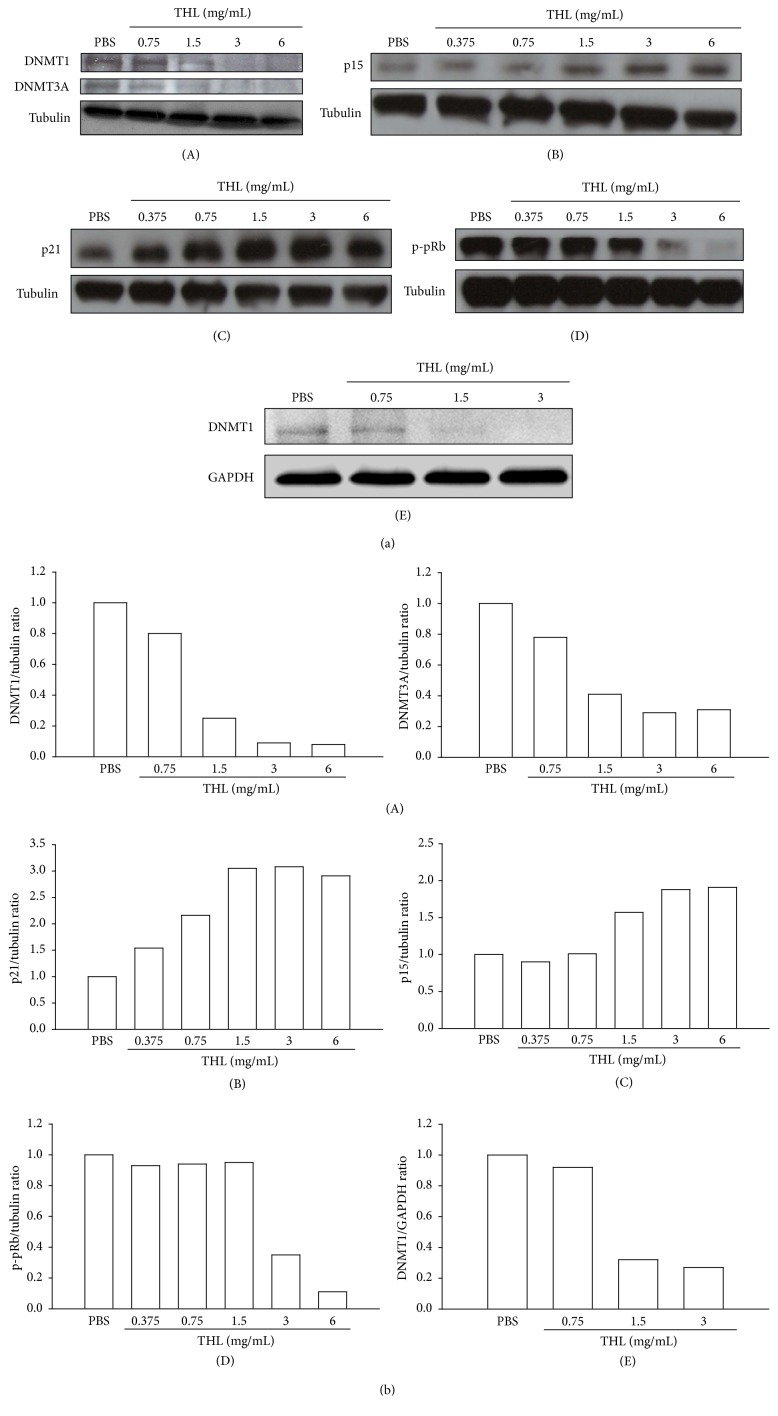
THL downregulated DNMT1 and DNMT3A accompanied by the induction of cyclin-dependent kinase inhibitors (p21 and p15) and the reduction of pRb phosphorylation in MCF-7 cells. THL also diminished the DNMT1 protein level in BT-474 cells. (a) (A) THL dose-dependently decreased the protein levels of DNMT1 and DNMT3A in MCF-7 cells. (B) THL dose-dependently increased the protein level of p21. (C) THL dose-dependently increased the protein level of p15. (D) THL dose-dependently decreased the phosphorylation (inactivation) of Rb protein (pRb) in MCF-7 cells. (E) THL dose-dependently diminished the protein level of DNMT1 in BT-474 cells. Cells were treated with PBS (control) or THL for 72 h and then harvested. Cell lysates were then prepared for Western blot analysis. (b) The blots were normalized to tubulin or GAPDH, and the fold change protein level expression is reported in comparison to PBS-treated control.

Previous study by Vijayaraghavalu et al. showed that treatment of MCF-7 cells with decitabine, DNMT inhibitor, resulted in G2/M cycle arrest and CDK inhibitor p21 induction [12]. Our previous work described the downregulation of DNMT1 by THL in APL cells [5]. Prompted by this finding and the observed THL-induced accumulation of MCF-7 cells in G2/M phase (Figure 2 and Table 1), we examined if THL also downregulated DNMT1 and induced p21 in MCF-7 cells. Indeed, THL significantly decreased DNMT1 protein level in a dose-dependent manner (Figure 4(A)). In addition to DNMT1, DNMT3A was also found to be elevated in sporadic breast cancer [11]. The protein level of DNMT3A was also reduced by THL (Figure 4(A)). The downregulation of DNMT1 was accompanied by a significant induction of p21 (Figure 4(B)) and another CDK inhibitor p15 (Figure 4(C)). Both expressions of p21 and p15 were shown to be repressed by DNMT1-mediated methylation [12, 21]. Accordingly, DNMT1 inhibition might play a central role in the anticancer effects of THL. Induction of CDK (cyclin-dependent kinase) inhibitors can prevent the inactivation (by phosphorylation) of the tumor suppressor effects of pRb, allowing it to restrain the progression of the cell cycle [22]. In accordance with the induction of CDK inhibitors such as p21 and p15, the phosphorylation of Rb protein (phospho-pRb) was markedly inhibited in THL-treated MCF-7 cells (Figure 4(D)). In addition to MCF-7 cells, the DNMT1-inhibitory effect mediated by THL was also observed in BT-474 cells (Figure 4(E)), which showed increase of SubG1 fraction and S-phase arrest in response to THL treatment (Table 3).

### 3.4. THL Suppresses AKT and ERK Signaling Pathways in MCF-7 Breast Cancer Cells

**Figure 5:**
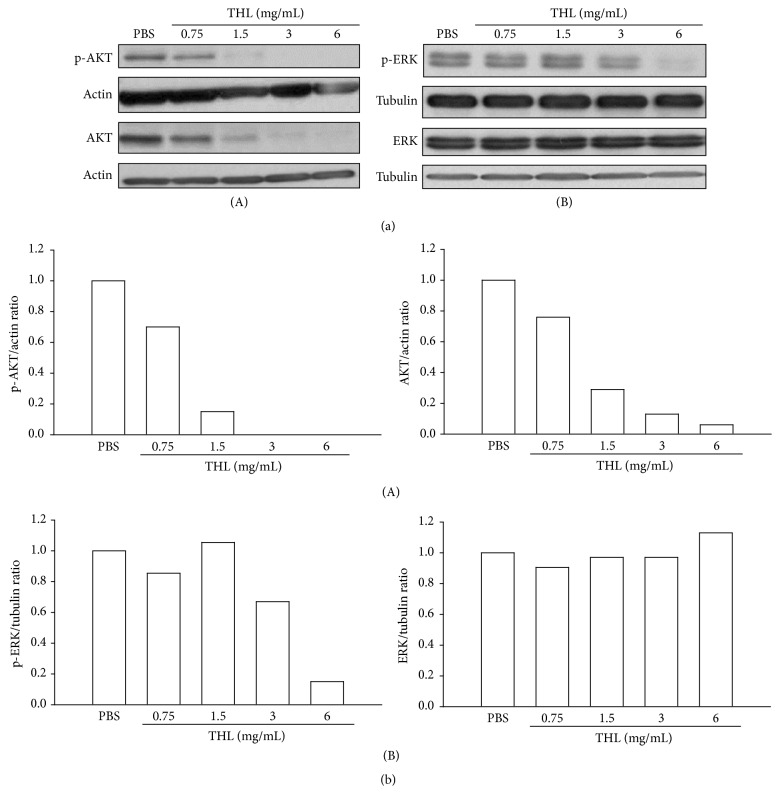
THL inhibited AKT and ERK signaling pathways in MCF-7 cells. (a) (A) THL decreased both the phosphorylated (p-AKT) and total AKT proteins in a dose-dependent manner. (B) THL decreased phosphorylated ERK protein (p-ERK) at doses above 1.5 mg/mL but did not affect the total ERK protein level. Cells were treated with PBS or THL for 72 h and then harvested. Cell lysates were then prepared for Western blot analysis. (b) The blots were normalized to actin or tubulin, and the fold change protein level expression is shown in comparison to PBS-treated control.

It is reported that AKT and ERK (extracellular signal-regulated kinase) signaling pathways are commonly dysregulated in human cancers, including breast cancer, leading to uncontrolled cellular proliferation [23]. We examined whether THL had any influence on these signaling pathways. As shown in Figure 5(A), both the phospho- and total forms of AKT protein were signally diminished by THL in a dose-dependent manner. Unlike its effect on AKT protein, THL reduced the phospho-ERK (p-ERK) only at doses >1.5 mg/mL but did not affect the total ERK protein level even at the highest dose tested (Figure 5(B)). THL appeared to substantially suppress AKT signaling of MCF-7 cells. At higher doses of THL, the ERK pathway was also observed to be inhibited.

### 3.5. THL Sensitizes MCF-7 Breast Cancer Cells to Radiation

**Figure 6:**
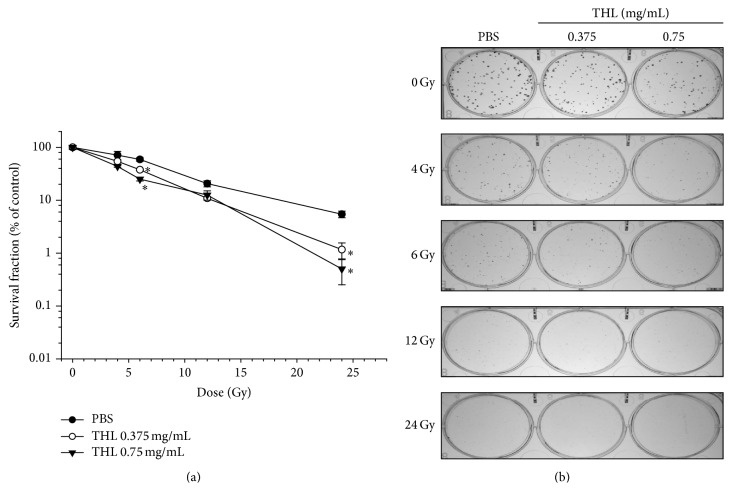
THL sensitizes MCF-7 cells to radiation. (a) Cells were pretreated with THL for 24 h and then irradiated with 4–24 Gy of ionizing radiation as indicated. Colony formation of the irradiated cells was assayed after 8 days of incubation. (b) Representative picture of colony formed by the cells described in (a). Data (mean ± SE in triplicate) are expressed as a percentage of the PBS control. ^*∗*^ *p* < 0.05, compared to the respective THL free group treated with the same dose of radiation.

Previous studies established that G2/M phase arrest [24] and DNMT1 inhibition [8, 13, 14] could sensitize cancer cells to radiation. Based on this observation, we examined the change in radiosensitivity of THL-treated MCF-7 cells. As shown in Figure 6(a), pretreatment with THL for 24 h enhanced radiation-mediated suppression of colony formation of MCF-7 cells. The dose of irradiation required for inhibiting colony formation by 50% in the control MCF-7 cells was 7.5 Gy. Pretreatment with THL at doses of 0.375 and 0.75 mg/mL reduced the dose required for 50% colony inhibition to 4.5 and 3.5 Gy, respectively. This corresponded to sensitizer enhancement ratios of 1.7 and 2.1, respectively (Figure 6(a)). Representative images of colony formation by THL-treated and THL-untreated cells are shown in Figure 6(b). At the same dose of radiation, fewer colonies were observed in the THL-pretreated MCF-7 cells compared to control cells pretreated with PBS only.

### 3.6. THL Suppresses DNA Double-Strand Break Repair Protein Rad51 in MCF-7 Breast Cancer Cells

**Figure 7:**
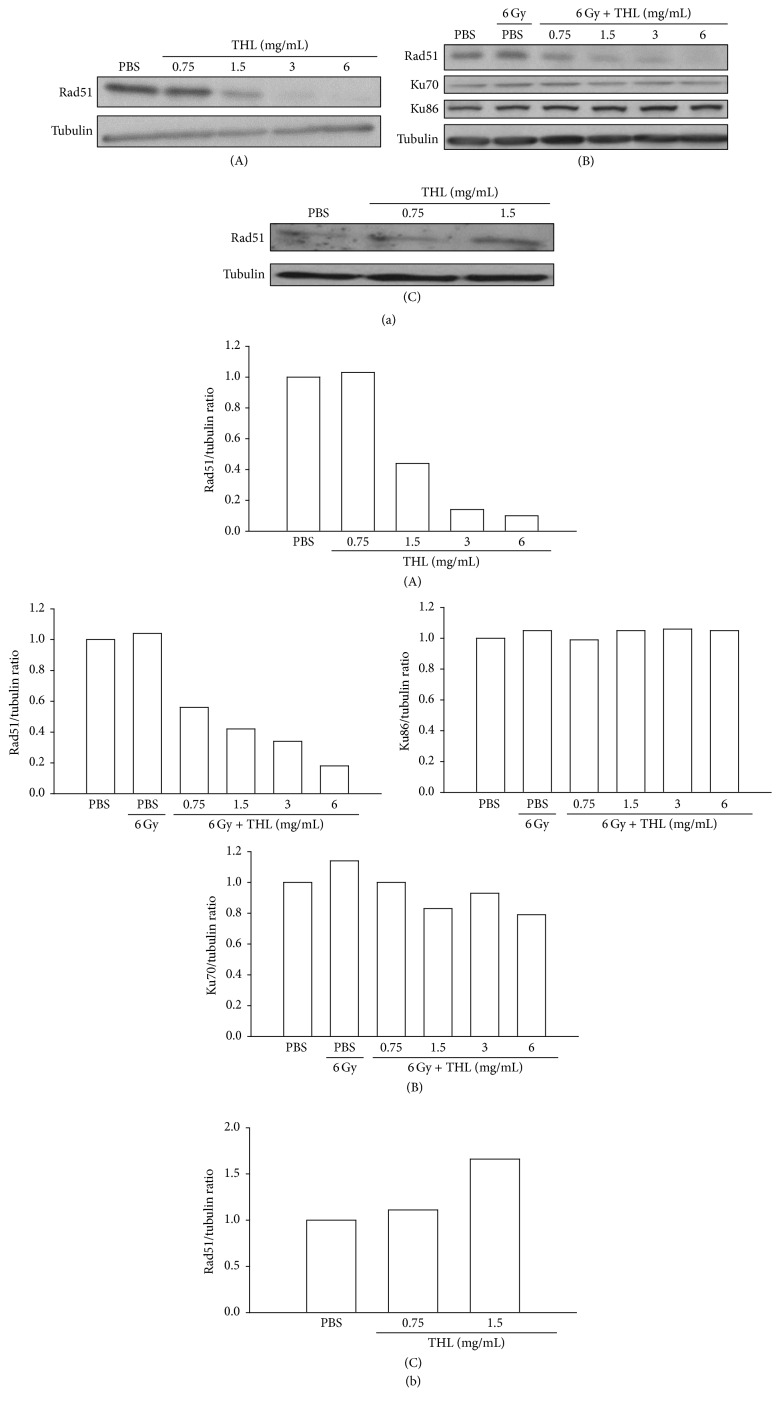
THL decreased DNA double-strand break repair protein Rad51 in MCF-7 cells but increased the level of the protein in primary HS68 fibroblast cells. (a) (A) THL dose-dependently decreased the homologous recombination DNA repair protein Rad51 in MCF-7 cells. (B) Pretreatment with THL dose-dependently decreased the Rad51 protein level in irradiated MCF-7 cells but did not reduce the nonhomologous end joining DNA repair proteins Ku70 and Ku86. (C) In contrast to its effect on MCF-7 cells, THL increased the Rad51 protein level in HS68 human foreskin fibroblast cells. In (A) and (C), cells were treated with PBS or THL for 72 h and then harvested. In (B), cells were pretreated with THL for 24 h and then irradiated with 6 Gy of ionizing radiation. Cells were harvested 24 h after irradiation, after which cell lysates were prepared for Western blot analysis. (b) The blots were normalized to actin or tubulin, and the fold change protein level expression is reported in comparison to PBS-treated control.

It was proposed that the radiosensitizing effects of DNMT1 inhibition were mediated by the inhibition of DNA DSB repair [13]. To investigate if this event was also associated with THL-mediated radiosensitization, we examined the DSB repair protein in THL-treated MCF-7 cells. As shown in Figure 7(A), the protein level of Rad51, a key homologous recombination DSB repair protein, was dose-dependently decreased by THL. In the 24 h THL-treated MCF-7 cells (prior to 6 Gy irradiation), the level of Rad51 protein was also significantly decreased in a dose-dependent manner; however, the levels of nonhomologous end joining DSB repair proteins such as Ku70 and Ku86 were not reduced (Figure 7(B)). These results suggested that inhibition of DSB repair was involved in THL-mediated radiosensitization in MCF-7 cells and inhibition of homologous recombination rather than nonhomologous end joining DSB repair might be responsible for this radiosensitizing effect. Importantly, THL did not reduce but instead increased the Rad51 protein level in HS68 human foreskin fibroblast cells (Figure 7(C)), which suggests that it would not increase the radiation sensitivity of normal cells

## 4. Discussion

The effect of THL on the downregulation of DNMT1 protein level has been shown in NB4 human APL cells in our previous work [5]. Presently, we demonstrate that THL also diminished the abnormally elevated DNMT1 accompanied by induction of p21 expression and G2/M cycle arrest in MCF-7 cells. This corroborates the findings of Vijayaraghavalu et al. for decitabine-treated MCF-7 [12]. Moreover, DNMT1 inhibition has been shown to radiosensitize cancer cells by suppressing DNA DSB repair activity [13]. In agreement with this observation, we found that THL enhanced the radiosensitivity of MCF-7 cells and reduced the DNA DSB repair protein Rad51. These results indicate that downregulation of DNMT1 may play an important role in THL-mediated anticancer effects.

In comparison with normal cells, the levels of DNMT1 are often aberrantly increased in cancer cells, causing transcriptional silencing of tumor suppressor genes by hypermethylation of their promoter CpG-rich regions [6]. The inhibition of DNMT1 as a means to reactivate the expression of these silenced tumor suppressors has been proposed as a rational strategy for cancer therapy [6, 9]. Nucleoside analogue DNMT inhibitors, such as 5-azacytidine (Vidaza) and 5-aza-2′-deoxycytidine (Dacogen, decitabine), have been approved by FDA as demethylating agents to treat myelodysplastic syndrome. Preclinical and clinical studies have been conducted to evaluate the applications of these two agents in cancer therapy [6, 12, 25]; however, their use is limited due to toxicity, stability issues, and the need for intravenous administration [26]. Therefore, the continued search for nonnucleoside DNMT1 inhibitors with greater safety and efficacy is warranted. As shown in this report, THL is herbal mixture that profoundly downregulated DNMT1 of cancer cells. This supports the use of THL as an adjunct for the complementary and integrative treatment of cancer. Indeed, a clinical trial has demonstrated the safety and immune augmentation efficacy of THL in breast cancer patients [1].

The downregulation of DNMT1 was also observed in THL-treated BT-474 cells with resultant S-phase arrest. Both MCF-7 and BT-474 have been shown to possess elevated DNMT1 protein levels [27]. This suggests that the DNMT1-inhibitory effect of THL is not specific to MCF-7 cells and that THL may be applied to reduce the viability of cancer cells with misregulated DNMT1 in general. Elevation of DNMT1 and inactivation of pRb are two ubiquitous features of cancer cells [28]. Subsequent studies showed that the pRb pathway regulates not only the expression [28] but also the stability [29] of DNMT1. In MCF-7 breast cancer cells, the elevated DNMT1 protein is caused by its abnormal stability [7]. The THL-induced decrease of DNMT1 levels in MCF-7 cells might be correlated with the reduced phosphorylation of pRb and the recovery of its activity. The exact details remain to be elucidated.

In addition to suppressing cancer cell proliferation [12], DNMT1 inhibition also sensitizes cancer cells to radiation [8, 13, 14]. A study by Kim et al. [13] showed that DNMT inhibitors such as decitabine, psammaplin A, and zebularine reduce DNMT1 and DNMT3A levels in cancer cells and prolong the expression of radiation-induced DSB marker *γ*H2AX, which is indicative of the impairment of DSB repair [13]. In alignment with this study, our results also show that THL reduces an important DNA DSB repair protein, Rad51. In mammalian cells, DNA DSBs are primarily repaired by homologous recombination and nonhomologous end joining, which are mediated mainly by Rad51 and Ku70/Ku86, respectively [30, 31]. The selective repression of Rad51 by THL might be beneficial for tumor specific radiosensitization, because homologous recombination, but not nonhomologous end joining DNA repair, is abnormally elevated in breast cancer cell lines, including MCF-7 cells [30]. The expression of Rad51 is tightly controlled in normal human cells; nevertheless, the majority of human tumor cells, including those of the breast, prostate, pancreas, lung, and cervix, overexpress Rad51 [32]. This overexpression is positively correlated with the aggressiveness and invasiveness of cancers and can also be used as a negative prognostic marker for patient survival time [32–34]. Therapies targeting Rad51 have been used to inhibit tumor growth and sensitize cancer cells to chemotherapies [32, 35], and Rad51-based therapy is likely to be associated with very low toxicity [32]. In support of this notion, THL does not decrease Rad51 in HS68 human primary foreskin fibroblast cells. The radiosensitizing effect of THL appears to be tumor specific. Based on the foregoing discussion, the role of Rad51 downregulation in THL-mediated anticancer effects deserves in-depth study.

Triple-negative breast cancers have poorer survival rates regardless of stage than those of other subtypes [36]. Due to the lack of expression of the estrogen receptor (ER) and human epidermal growth factor receptor 2 (HER2), this type of breast cancer cannot be effectively treated by hormone and targeted therapies in the form of antiestrogens (e.g., tamoxifen) and monoclonal antibodies [e.g., trastuzumab (Herceptin)], respectively [36]. The strategy to treat triple-negative breast cancers by targeting DNMT1 has been proposed recently [37, 38]. Consistent with this proposition, Chia et al. showed that THL inhibits the growth of human MDA-MB-231 (triple-negative type) breast cancer xenografts [4]. The use of THL in a complementary setting may benefit patients who are suffering from triple-negative breast cancer.

A recent study by Pathania et al. showed that inhibition of DNMT1 suppresses the cancer stem cell (CSC) population in breast cancer cells [39]. Our previous work also demonstrated that THL eliminates the CSC-like “side population” cells in hepatoma cell lines [40]. Therefore, inhibition of DNMT1 may be an important mechanism underlying the THL-mediated suppression of the CSC population in cancer cells. As the existence of CSC population and drug resistance are the main obstacles in successful cancer therapy, the complementary use of THL may be helpful to overcome the difficulties of current treatments in breast cancer patients. Additionally, inhibition of DNMT1 [12, 41] or elimination of Rad51 [35] has been shown to sensitize cancer cells to chemotherapeutic agents. As the current study suggests, the pharmacological effects THL on DNMT1 and Rad51 are consistent with this favorable chemotherapeutic outcome. FDA-approved randomized phase II clinical trial will be conducted soon in refractory breast cancer patients to evaluate the clinical benefits of THL in combination with chemotherapy.

## 5. Conclusion

Our results demonstrate that THL induces G2/M cell cycle arrest and radiosensitization in MCF-7 cells. The main underlying mechanisms are proposed to involve tumor suppressor (p21 and p15) induction and DNA repair repression orchestrated by the downregulation of DNMT1. In order for the complete integration of THL into complementary cancer therapy regimens, a comprehensive and detailed investigation of its mechanisms of action is warranted, coupled with thorough efficacy assessments through clinical trials.

## Figures and Tables

**Table 1:** Cell cycle phase distribution (%) of 72-hour THL-treated MCF-7 cells showing G2/M arrest.

Treatment	SubG1 (%)	Cell cycle distribution		
		G1 (%)	S (%)	G2/M (%)
PBS	0.5	67.45	9.6	22.95
THL 0.75 mg/mL	1.53	68.37	6.87	24.75
THL 1.5 mg/mL	1.23	59.54	7.05	33.41
THL 3 mg/mL	1.16	44.93	10.74	44.33
THL 6 mg/mL	0.52	40.60	13.56	45.84

**Table 2:** Cell cycle phase distribution (%) of 72-hour THL-treated MDA-MB-231 cells showing G2/M arrest.

Treatment	SubG1 (%)	Cell cycle distribution		
		G1 (%)	S (%)	G2/M (%)
PBS	0.6	50.4	22.6	27.1
THL 0.75 mg/mL	0.5	48.8	22.5	28.7
THL 1.5 mg/mL	0.6	50.5	21.7	27.8
THL 3 mg/mL	2.3	47.7	20.4	31.9
THL 6 mg/mL	2.1	28.2	19.3	52.5

**Table 3:** Cell cycle phase distribution (%) of 72-hour THL-treated BT-474 cells showing increasing SubG1 fraction and S-phase arrest.

Treatment	SubG1 (%)	Cell cycle distribution		
		G1 (%)	S (%)	G2/M (%)
PBS	0.8	72.2	12.8	15.0
THL 0.75 mg/mL	3.8	67.5	16.7	15.8
THL 1.5 mg/mL	13.5	65.8	17.7	16.6
THL 3 mg/mL	27.3	55.8	27.7	16.5
THL 6 mg/mL	50.1	50.7	36.9	12.4

## Abbreviations

APL: Acute promyelocytic leukemia
CDK: Cyclin-dependent kinase
DNMT1: DNA methyltransferase 1
DNMT3A: DNA methyltransferase 3A
GAPDH: Glyceraldehyde-3-phosphate dehydrogenase
PBS: Phosphate-buffered saline
SE: Standard error
SRB: Sulforhodamine B
THL: Tien-Hsien Liquid.

## References

[1] Kuo W.-H., Yao C.-A., Lin C. H., Chang K.-J. (2012). Safety and efficacy of Tien-Hsien Liquid Practical in patients with refractory metastatic breast cancer: a randomized, double-blind, placebo-controlled, parallel-group, phase IIa trial. *Evidence-Based Complementary and Alternative Medicine*.

[2] Sun A., Chia J.-S., Wang W.-B., Chiang C.-P. (2004). Immunomodulating effects of ‘Tien-Hsien liquid’ on peripheral blood mononuclear cells and T-lymphocytes from patients with recurrent aphthous ulcerations. *American Journal of Chinese Medicine*.

[3] Sun A., Chia J.-S., Chiang C.-P. (2005). The Chinese herbal medicine Tien-Hsien liquid inhibits cell growth and induces apoptosis in a wide variety of human cancer cells. *Journal of Alternative and Complementary Medicine*.

[4] Chia J.-S., Du J.-L., Hsu W.-B., Sun A., Chiang C.-P., Wang W.-B. (2010). Inhibition of metastasis, angiogenesis, and tumor growth by Chinese herbal cocktail Tien-Hsien Liquid. *BMC Cancer*.

[5] Lai T.-Y., Yao C.-J., Yang C.-M. (2011). Targeting PML-RAR*α* and oncogenic signaling pathways by Chinese herbal mixture *Tien-Hsien* liquid in acute promyelocytic leukemia NB4 cells. *Evidence-Based Complementary and Alternative Medicine*.

[6] Subramaniam D., Thombre R., Dhar A., Anant S. (2014). DNA methyltransferases: a novel target for prevention and therapy. *Frontiers in Oncology*.

[7] Agoston A. T., Argani P., Yegnasubramanian S. (2005). Increased protein stability causes DNA methyltransferase 1 dysregulation in breast cancer. *The Journal of Biological Chemistry*.

[8] Zielske S. P. (2015). Epigenetic dna methylation in radiation biology: on the field or on the sidelines?. *Journal of Cellular Biochemistry*.

[9] Singh V., Sharma P., Capalash N. (2013). DNA methyltransferase-1 inhibitors as epigenetic therapy for cancer. *Current Cancer Drug Targets*.

[10] Pellerito C., Morana O., Ferrante F. (2015). Synthesis, chemical characterization, computational studies and biological activity of new DNA methyltransferases (DNMTs) specific inhibitor. Epigenetic regulation as a new and potential approach to cancer therapy. *Journal of Inorganic Biochemistry*.

[11] Yu Z., Xiao Q., Zhao L. (2015). DNA methyltransferase 1/3a overexpression in sporadic breast cancer is associated with reduced expression of estrogen receptor-alpha/breast cancer susceptibility gene 1 and poor prognosis. *Molecular Carcinogenesis*.

[12] Vijayaraghavalu S., Dermawan J. K., Cheriyath V., Labhasetwar V. (2013). Highly synergistic effect of sequential treatment with epigenetic and anticancer drugs to overcome drug resistance in breast cancer cells is mediated via activation of p21 gene expression leading to G2/M cycle arrest. *Molecular Pharmaceutics*.

[13] Kim H. J., Kim J. H., Chie E. K., Da Young P., Kim I. A., Kim I. H. (2012). DNMT (DNA methyltransferase) inhibitors radiosensitize human cancer cells by suppressing DNA repair activity. *Radiation Oncology*.

[14] Qiu H., Yashiro M., Shinto O., Matsuzaki T., Hirakawa K. (2009). DNA methyltransferase inhibitor 5-aza-CdR enhances the radiosensitivity of gastric cancer cells. *Cancer Science*.

[15] Leigh A. B., Cheung H. P., Lin L. Z. Comprehensive and holistic analysis of HT-29 colorectal cancer cells and tumor-bearing nude mouse model: interactions among fractions derived from the Chinese medicine formula Tian Xian liquid in effects on human colorectal carcinoma.

[16] Skehan P., Storeng R., Scudiero D. (1990). New colorimetric cytotoxicity assay for anticancer-drug screening. *Journal of the National Cancer Institute*.

[17] Vichai V., Kirtikara K. (2006). Sulforhodamine B colorimetric assay for cytotoxicity screening. *Nature Protocols*.

[18] Hsieh W.-T., Huang K.-Y., Lin H.-Y., Chung J.-G. (2006). Physalis angulata induced G2/M phase arrest in human breast cancer cells. *Food and Chemical Toxicology*.

[19] Clemens D. L., Calisto L. E., Sorrell M. F., Tuma D. J. (2003). Ethanol metabolism results in a G2/M cell-cycle arrest in recombinant Hep G2 cells. *Hepatology*.

[20] Smits V. A. J., Klompmaker R., Vallenius T., Rijksen G., Mäkelä T. P., Medema R. H. (2000). p21 Inhibits Thr161 phosphorylation of Cdc2 to enforce the G2 DNA damage checkpoint. *The Journal of Biological Chemistry*.

[21] Yu J., Peng Y., Wu L.-C. (2013). Curcumin down-regulates DNA methyltransferase 1 and plays an anti-leukemic role in acute myeloid leukemia. *PLoS ONE*.

[22] Knudsen E. S., Knudsen K. E. (2008). Tailoring to RB: tumour suppressor status and therapeutic response. *Nature Reviews Cancer*.

[23] Saini K. S., Loi S., de Azambuja E. (2013). Targeting the PI3K/AKT/mTOR and Raf/MEK/ERK pathways in the treatment of breast cancer. *Cancer Treatment Reviews*.

[24] Choy H., Rodriguez F. F., Koester S., Hilsenbeck S., Von Hoff D. D. (1993). Investigation of taxol as a potential radiation sensitizer. *Cancer*.

[25] Thummuri D., Kumar S., Surapaneni S. K., Tikoo K. (2015). Epigenetic regulation of protein tyrosine phosphatase PTPN12 in triple-negative breast cancer. *Life Sciences*.

[26] Billam M., Sobolewski M. D., Davidson N. E. (2010). Effects of a novel DNA methyltransferase inhibitor zebularine on human breast cancer cells. *Breast Cancer Research and Treatment*.

[27] Menschikowski M., Hagelgans A., Nacke B., Jandeck C., Sukocheva O., Siegert G. (2015). Epigenetic control of phospholipase A2 receptor expression in mammary cancer cells. *BMC Cancer*.

[28] McCabe M. T., Davis J. N., Day M. L. (2005). Regulation of DNA methyltransferase 1 by the pRb/E2F1 pathway. *Cancer Research*.

[29] Agoston A. T., Argani P., De Marzo A. M., Hicks J. L., Nelson W. G. (2007). Retinoblastoma pathway dysregulation causes DNA methyltransferase 1 overexpression in cancer via MAD2-mediated inhibition of the anaphase-promoting complex. *The American Journal of Pathology*.

[30] Mao Z., Jiang Y., Liu X., Seluanov A., Gorbunova V. (2009). DNA repair by homologous recombination, but not by nonhomologous end joining, is elevated in breast cancer cells. *Neoplasia*.

[31] Wang J., Liu Q., Yang Q. (2012). Radiosensitization effects of berberine on human breast cancer cells. *International Journal of Molecular Medicine*.

[32] Hine C. M., Seluanov A., Gorbunova V. (2012). Rad51 promoter-targeted gene therapy is effective for in vivo visualization and treatment of cancer. *Molecular Therapy*.

[33] Nogueira A., Catarino R., Coelho A., Araújo A., Gomes M., Medeiros R. (2010). Influence of DNA repair RAD51 gene variants in overall survival of non-small cell lung cancer patients treated with first line chemotherapy. *Cancer Chemotherapy and Pharmacology*.

[34] Maacke H., Opitz S., Jost K. (2000). Over-expression of wild-type Rad51 correlates with histological grading of invasive ductal breast cancer. *International Journal of Cancer*.

[35] Du L.-Q., Wang Y., Wang H., Cao J., Liu Q., Fan F.-Y. (2011). Knockdown of Rad51 expression induces radiation- and chemo-sensitivity in osteosarcoma cells. *Medical Oncology*.

[36] Bauer K. R., Brown M., Cress R. D., Parise C. A., Caggiano V. (2007). Descriptive analysis of estrogen receptor (ER)-negative, progesterone receptor (PR)-negative, and HER2-negative invasive breast cancer, the so-called triple-negative phenotype: a population-based study from the California Cancer Registry. *Cancer*.

[37] Choi D. S., Blanco E., Kim Y.-S. (2014). Chloroquine eliminates cancer stem cells through deregulation of Jak2 and DNMT1. *Stem Cells*.

[38] Tang H., Liu P., Yang L. (2014). miR-185 suppresses tumor proliferation by directly targeting E2F6 and DNMT1 and indirectly upregulating BRCA1 in triple-negative breast cancer. *Molecular Cancer Therapeutics*.

[39] Pathania R., Ramachandran S., Elangovan S. (2015). DNMT1 is essential for mammary and cancer stem cell maintenance and tumorigenesis. *Nature Communications*.

[40] Yao C.-J., Yeh C.-T., Lee L.-M. (2012). Elimination of cancer stem-like side population cells in hepatoma cell lines by Chinese herbal mixture Tien-Hsien Liquid. *Evidence-Based Complementary and Alternative Medicine*.

[41] Li S.-Y., Sun R., Wang H.-X. (2015). Combination therapy with epigenetic-targeted and chemotherapeutic drugs delivered by nanoparticles to enhance the chemotherapy response and overcome resistance by breast cancer stem cells. *Journal of Controlled Release*.

